# Histone methyltransferases SDG33 and SDG34 regulate organ-specific nitrogen responses in tomato

**DOI:** 10.3389/fpls.2022.1005077

**Published:** 2022-10-12

**Authors:** Carol Bvindi, Liang Tang, Sanghun Lee, Ryan M. Patrick, Zheng Rong Yee, Tesfaye Mengiste, Ying Li

**Affiliations:** ^1^ Department of Botany and Plant Pathology, Purdue University, West Lafayette, IN, United States; ^2^ Purdue Center for Plant Biology, Purdue University, West Lafayette, IN, United States; ^3^ Department of Horticulture and Landscape Architecture, Purdue University, West Lafayette, IN, United States

**Keywords:** histone methyltransferases, nitrogen responses, chromatin modifications, functional genomics, network analysis

## Abstract

Histone posttranslational modifications shape the chromatin landscape of the plant genome and affect gene expression in response to developmental and environmental cues. To date, the role of histone modifications in regulating plant responses to environmental nutrient availability, especially in agriculturally important species, remains largely unknown. We describe the functions of two histone lysine methyltransferases, SET Domain Group 33 (SDG33) and SDG34, in mediating nitrogen (N) responses of shoots and roots in tomato. By comparing the transcriptomes of CRISPR edited tomato lines *sdg33* and *sdg34* with wild-type plants under N-supplied and N-starved conditions, we uncovered that SDG33 and SDG34 regulate overlapping yet distinct downstream gene targets. In response to N level changes, both SDG33 and SDG34 mediate gene regulation in an organ-specific manner: in roots, SDG33 and SDG34 regulate a gene network including *Nitrate Transporter 1.1* (*NRT1.1*) and *Small Auxin Up-regulated RNA* (*SAUR*) genes. In agreement with this, mutations in *sdg33* or *sdg34* abolish the root growth response triggered by an N-supply; In shoots, SDG33 and SDG34 affect the expression of photosynthesis genes and photosynthetic parameters in response to N. Our analysis thus revealed that SDG33 and SDG34 regulate N-responsive gene expression and physiological changes in an organ-specific manner, thus presenting previously unknown candidate genes as targets for selection and engineering to improve N uptake and usage in crop plants.

## Introduction

Nitrogen (N) is a rate-limiting macronutrient for plants (Ueda et al., 2017). The application of N fertilizer has greatly contributed to the yield increase during the green revolution, however, it has also aggravated environmental problems including eutrophication and climate change (Munoz-Huerta et al., 2013). To promote agricultural productivity and sustainability, a comprehensive understanding of N sensing, uptake, and utilization is essential. Plants uptake N from the soil mainly in the form of nitrate ( $NO_{3}^{-}$ ) and ammonium ( $NH_{4}^{+}$ ), which once inside plant cells are reduced and assimilated into amino acids. Nitrate is mobile in the soil, therefore, the availability of N in soil is highly variable at both spatial and temporal scales. To maximize N uptake and assimilation, plants respond to changing N conditions through adequate morphological and metabolic changes (Masclaux-Daubresse et al., 2010; Vidal et al., 2020). One remarkable example of morphological plasticity is that plants alter their root architecture to effectively exploit the available N in the soil. N abundance in the soil often promotes lateral root proliferation to increase N uptake capacity (Remans et al., 2006; Zhang et al., 2007; Lima et al., 2010; Xie et al., 2014), whereas N deficient conditions promote primary/axial root elongation (Yoneyama et al., 2012; Zhang et al., 2012; Sun et al., 2014). While these morphological responses occur in days, metabolic changes could be in place within minutes or hours after N level changes. Metabolic responses to N availability include modulation of nitrate/ammonium transporter activities (Sonoda et al., 2003; Gu et al., 2013), regulation of N assimilation enzymes, and higher carbon assimilation rate in response to higher N content (Xu et al., 2012; Griffiths et al., 2016). Moreover, N status was shown to affect plant defense responses (Verly et al., 2020; Soulie et al., 2020). These morphological and metabolic responses often involve the regulation of relevant genes and gene networks. Genes involved in the N responses have been well characterized and their expression is controlled by complex gene regulatory networks (Vidal et al., 2014; Ueda et al., 2017; Zhao et al., 2018). Multiple transcription factors, e.g. HRS1 (Kiba et al., 2018), TGAs (Alvarez et al., 2014), NLP (Guan et al., 2017; Alvarez et al., 2020), and bZIPs (Yang et al., 2019), have been characterized as important regulators of N signaling and metabolism.

In recent years, chromatin regulatory mechanism has emerged as an integral player in gene regulation during environmental responses (Zhao et al., 2005; Secco et al., 2017; Friedrich et al., 2019; Chang et al., 2020). Histone lysine methylation, a specific form of chromatin modification, depicts covalent modifications of lysine residues of histone subunits H3 and H4 by methyl groups. Histone lysine methylation is catalyzed by histone lysine(K) methyltransferases (HKMTs) of the SET domain group (SDGs) protein family (Makarevich et al., 2006). Histone lysine methylation influences chromatin structure and accessibility of the associated genomic DNA to transcriptional machinery, therefore modulating gene expression (Kouzarides, 2007; Thorstensen et al., 2008; Alvarez et al., 2010). Different levels of methylation (mono-, di- or tri-) at different lysine residues can be associated with either activation or repression of gene expression, thus adding to the complexity of gene regulation (Upadhyay and Cheng, 2011). Recent efforts, mostly in the model plant Arabidopsis, have begun to reveal the role of chromatin regulation in mediating N responses. In Arabidopsis, the regulation of high-affinity transporter *NITRATE TRANSPORTER 2.1* (*NRT2.1*) was reported to be associated with the increase of repressive histone methylation mark H3K27me3 and decrease of permissive marks H3K4me3 and H3K36me3 (Widiez et al., 2011). At the genome-wide level, a histone methyltransferase SDG8 was shown to mediate epigenetic and transcriptional regulation of N-responsive genes in Arabidopsis (Li et al., 2019). However, in contrast to our knowledge of N-relevant chromatin modifications in Arabidopsis, our understanding of chromatin regulation of nutrient responses in crop species, which have been bred with supplemented N, remains rather scarce. In addition, while previous studies largely focused on whole seedlings, the role of histone modification in controlling root plasticity in response to changing N has rarely been explored.

In this study, we address these knowledge gaps by presenting that two tomato histone lysine methyltransferases SDG33 (Solyc04g057880.2.1) and SDG34 (Solyc06g059960.2.1), homologs of Arabidopsis SDG8 (Aiese Cigliano et al., 2013), mediate the expression of gene networks and physiological changes in response to N signals in the shoots and roots. In Arabidopsis, SDG8 functions as H3K36 methyltransferase to regulate multiple metabolic processes and environmental responses, including nitrogen response (Zhao et al., 2005; Xu et al., 2008; Berr et al., 2010; Li et al., 2015; Lee et al., 2016; Jiang et al., 2018; Li et al., 2020), and is highly conserved among eukaryotes, with homologs in human (Li et al., 2007), yeast (Krogan et al., 2003), rice (Liu et al., 2016), and tomato (Aiese Cigliano et al., 2013). The tomato homologs of SDG8, SDG33 and SDG34, are also shown to affect H3K4 and H3K36 methylation (Bvindi et al., 2022). We test the hypothesis that the function of SDG8 in mediating N responses is conserved across species and that the tomato SDG33 and/or SDG34 also regulate N response. Through transcriptomic profiling of CRISPR edited *sdg33* and *sdg34* mutants under N-treatments, we uncover that SDG33 and SDG34 control N-responsive gene regulatory networks in an organ-specific manner. Specifically, in the roots SDG33 and SDG34 regulate *NITRATE TRANSPORTER 1.1* (*NRT1.1*) and *SMALL AUXIN UP-REGULATED RNA* (*SAUR*), as well as N-responsive root growth, thus suggesting histone methylation as the possible epigenetic mechanisms for N-mediated root plasticity. Moreover, the two paralogous SDG33 and SDG34 regulate shared downstream gene networks, as well as control distinct downstream genes, thus providing insights into the functional divergence of histone methyltransferases.

## Materials and methods

### Plant growth conditions and treatments

CRISPR mutants *sdg33* and *sdg34* created previously in our labs (Bvindi et al., 2022) and the corresponding wild type (WT) tomato (*Solanum lycopersicum*) cultivar CastlemartII, a widely grown cultivated tomato cultivar, was used in this study. Specifically, *sdg33.1* (referred to as *sdg33-64B* in Bvindi et al., 2022), *sdg33.2* (a.k.a *sdg33-64S*), *sdg34.1* (*sdg34-76*), and *sdg34.2* (*sdg34-27*) were used, while *sdg33.1* and *sdg34.1* were used for transcriptome specifically. Seeds were surface sterilized with 20% sodium hypochlorite for 20 minutes and rinsed with distilled water. The seeds were then germinated on filter paper in the dark until the radicle emerged and transferred into black sand for the seedlings to be established for one week. Seedlings were then grown hydroponically in foil tapped plastic containers with 1L nutrient medium, which consisted of 1.2 mM KNO_3_, 0.8mM Ca(NO_3_)_2_, 0.2mM NH_4_H_2_PO_4_, 0.2mM MgSO4, 50μM KCl, 12.5 μM H_3_BO_3_, 1 μM MnSO_4_, 1 μMZnSO_4_, 0.5 μMCuSO_4_, 0.1 μM H_2_MoO_4_, 0.1 μM NiSO_4_ and 10 μM Fe-EDDHA (Wang et al., 2001). Plants were grown in the greenhouse with a photoperiod of 14-h light and 10-h dark at 24°C. Two plants were grown in one container and aeration was provided for one hour daily to increase the oxygen content of the nutrient medium. The nutrient medium was refreshed every three days.

After two weeks of growth in the nutrient medium, plants were transferred into the starvation medium, which is identical to the nutrient medium except that 1.2 mM KNO_3_, 0.8mM Ca(NO_3_)_2_, 0.2mM NH_4_H_2_PO_4_ were replaced by 0.6mM K_2_SO_4_, 0.8mM CaSO_4_, 0.2mM KH_2_PO_4_ respectively. After two days in the starvation medium, plants were transferred into two treatment media: (1) with N (+N); and (2) without N as control (-N). The +N treatment medium was the same as the growth medium and the -N treatment medium was the same as the starvation solution. Plants were grown in respective treatments for five hours before being harvested for RNA analysis. Plants were grown in respective treatments for seven days and phenotyped on the eighth day for root architecture, fresh shoot weight, and chlorophyll content.

### RNA extraction

Plants were grown hydroponically as described above. Three independent plants were pooled to make one replication and three replications per treatment per genotype were used for RNA extraction. RNA for transcriptome profiling was extracted from the root and shoot samples harvested five hours after N treatments. Total RNA was extracted from the shoots and roots using Trizol (Invitrogen) according to the manufacturer’s instructions. After extraction, total RNA was treated with DNase (New England Biolabs) according to the manufacturer’s instructions. RNA was then precipitated with 3M sodium acetate and three-times volume of 100% ethanol and resuspended in DEPC treated water. RNA was then concentrated using ZYMO RNA clean and concentrator kit (ZYMO Research) according to the manufacturer’s instructions. The integrity of RNA was accessed by the Agilent Bioanalyzer (Agilent technologies).

### RNA sequencing and data analysis

RNA samples were submitted to the Purdue Genomics Core for RNA-Seq library preparation and sequencing on an Illumina Novaseq platform with paired-end 50bp format. On average, approximately 25 million read pairs were generated for each library. The raw sequencing reads were trimmed by the Purdue Genomics Core to remove adaptors and low-quality reads. Next, trimmed reads were mapped against the tomato genome (build 3.0, assessed on June 5, 2019) using BBmap (Bushnell et al., 2017). Next, mapped reads were used to generate a count for each gene feature in the genome, using the tomato gene models ITAG3.2 and the annotated miRNA loci based on miRbase (Kozomara and Griffiths-Jones, 2011), using FeatureCounts (Liao et al., 2014). The expression level determined by RNA-seq agree well with that determined by qRT-PCR (**Figure S1**). Finally, differentially expressed genes were detected using DESeq2 (Love et al., 2014) with the design (~Genotype+Treatment+Genotype : Treatment), for *sdg33* and *sdg34*, separately. The GO analyses for the DEGs were first performed using AgriGO (Du et al., 2010) database with FDR < 0.05 unless otherwise noted (*e.g.* by ShinyGO (Ge et al., 2020)). Then the enrichment results were further processed using the ReviGO tool (Supek et al., 2011) which clusters enriched GO terms based on semantic similarity to reduce the redundant GO terms and prioritize the most representative ones. The ReviGO analysis was performed using default parameters, with the allowed similarity being 0.9. The heatmaps and clustering were performed using Mev (Howe et al., 2011) and the heatmaps show the normalized expression levels (by full quantile method in EDAseq) which was then normalized to the mean of the row for heatmap.

### Network module analysis

To perform the network analysis, the gene count matrix containing 36 RNA-seq libraries (3 reps x 3 genotypes x 2 treatments =18 libraries from the root samples and 18 libraries from the shoot samples) was normalized by a median of ratios method using DEseq2. Next, the normalized gene counts were log-transformed (log2(x+1)) and filtered for low expression genes with the cutoff (mean gene count > 3). Next, the filtered expression matrix from shoots and roots were processed using the weighted gene co-expression network analysis (WGCNA) package (Langfelder and Horvath, 2008) to identify co-expression network modules with the following parameters: (1) soft-threshold power β = 9 to reach the scale-free network topology (model fitting index R2 > 0.8); and (2) dynamic tree cut for module identification (deepSplit =2 and minModuleSize =50). To identify the modules that were significantly enriched with the identified DEGs, the hypergeometric test was used to compare DEGs with genes in each module. To overlay the multinetwork interaction information from the model plant Arabidopsis, first, BLASTP was used to determine Arabidopsis homologous genes at an E-value cutoff of 1E-6. The homologous Arabidopsis genes corresponding to a network module were uploaded to the VirtualPlant platform to construct a network using “network analysis” tool by inquiring multinetwork database which comprises previously identified protein-protein interactions, enzymes, and metabolic information from the KEGG database, and transcription factor-gene interactions (Katari et al., 2010). The resultant gene networks were visualized and analyzed using Cytoscape (Shannon et al., 2003). Finally, the “hub” genes (with the largest number of outdegree) and the most regulated gene (with the largest number of indegree) inside each network module were selected using the NetworkAnalyzer function of the Cytoscape. To determine which GO categories are statistically overrepresented in the identified gene networks, the BiNGO (the biological networks Gene Ontology) plugin in the Cytoscape was used to perform GO term enrichment analysis.

### Chlorophyll measurement

Shoots were harvested at the end of the treatments and frozen in liquid nitrogen. The shoot tissue was ground to fine powder, weighed and chlorophyll was extracted with methanol by incubating at room temperature for 10 min with gentle rotation. The extract was centrifuged at 13000rpm for seven minutes and the supernatants were used for absorbance measurement. The absorbance was measured using supernatant at 750, 665, and 652nm by a Tecan microplate reader (Tecan Switzerland), using methanol as the blank. Chlorophyll a (Chl a), Chlorophyll b (Chl b), the ratio of Chl a:b, and total chlorophyll were calculated according to (J.Porra, 1990; Aiese Cigliano et al., 2013).

### Photosynthesis measurement

Plants were grown in standard greenhouse condition (14 h day/8h night, 24°C). Seeds were first germinated in clay for a week, and then grown by treating with the nutrient solutions (+N) twice a week for five weeks so that the leaves were big enough for photosynthetic measurements. Next, half of the plants were treated with +N solution and the other half were treated with -N solutions for a week, and then photosynthetic parameters were measured using the PhotoSynQ system (MultispecQ V2.0).

### Root architecture measurements

Roots grown in hydroponics solutions were laid as a single pane in a transparent tray with a thin layer of water placed on top of a lightbox and imaged by a fixed-height digital camera. Images were uploaded in the root imaging software GiAroots (Galkovskyi et al., 2012) for processing and phenotyping. The GiAroot pipeline consists of image preparation steps (scaling, rotating, and cropping), creating a greyscale image, and applying double adaptive imaging thresholding with pre-set parameters to produce binary foreground (root) or background (non-root). The binary images were then processed for all the root architecture trait calculations. Pixels were scaled to the appropriate dimension; millimeters, square millimeters or cubic millimeter’s using a reference ruler in the image.

### Statistical analysis of physiological traits

Two-way ANOVA was performed to test the impact of genotype, N treatment, and their interaction on each phenotypic trait. ANOVA was performed in the R software, version 3.3.1 (R Core Team, 2021) using the Agricolae package. Statistical significance was determined at the level P≤ 0.05. To further analyze which means are significantly different, means were compared using the student ‘s T-test. N response was also determined by comparing the means in the N treatment (+N) to the control treatment (-N) using a two-sample t-test assuming unequal variance.

## Results

### SDG33 and SDG34 regulate the expression of overlapping yet distinct downstream genes

**Figure 1:**
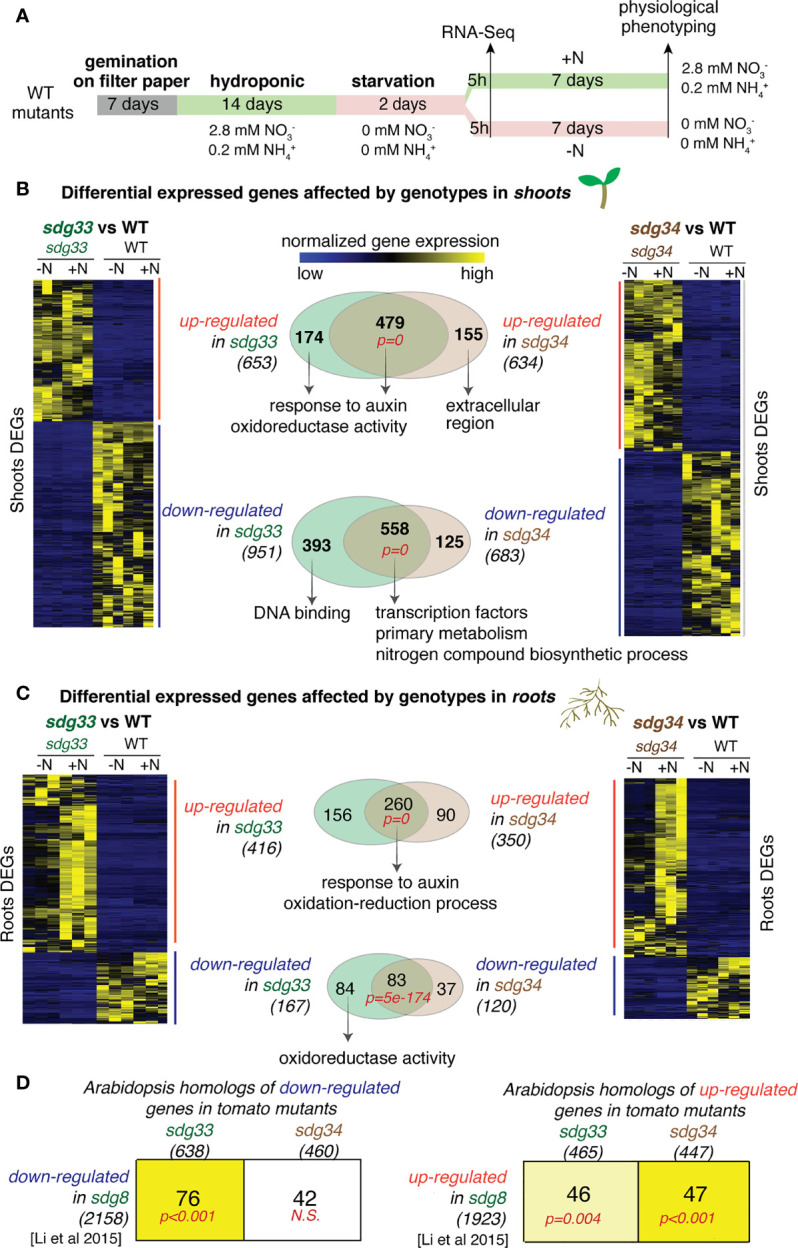
Differentially expressed genes (DEGs) that are mis-expressed in the *sdg33* or *sdg34* mutants were identified for shoots or roots and compared with the genes affected by the Arabidopsis homolog SDG8. **(A)** Experimental scheme of plant growth, treatment, transcriptome and phenotyping were shown. (B & C) Shoot and root transcriptomes were analyzed to identify DEGs that are affected by mutations in *SDG33* or *SDG34* regardless of the nitrogen conditions. The transcript levels of identified DEGs are shown as heatmaps. The Venn diagrams show the comparison of DEGs between *sdg33* and *sdg34*, for up-regulated and down-regulated genes separately, in shoots **(B)** and roots **(C)**. The p-values in red represents the statistical significance of observing the overlap between the DEGs in *sdg33* and that in *sdg34*, determined by hypergeometric distribution against the whole gene set as background. The numbers in parentheses are the total number of genes identified for each category, and the representative significantly enriched GO terms were listed for the commonly shared genes, or genes uniquely regulated by SDG33 or SDG34. **(D)** The DEGs identified in tomato shoots were first converted to their corresponding Arabidopsis homologs (shown in the columns) and then compared with the DEGs identified in Arabidopsis seedlings mutated in *sdg8* (the homolog of SDG33 and SDG34) (Li et al., 2015) (shown in the row). The size of each gene set was shown as the number within parentheses. Each cell includes the number of genes overlapped between the gene set represented by the column (DEGs from tomato) and the gene set represented by the row (DEGs from Arabidopsis). The p-values represent the significance of such overlaps determined by the Genesect function in the VirtualPlant platform. The bright yellow background of the cell indicates a highly significant overlap between the two gene sets (*i.e.* the overlap is significantly higher than what is expected by chance at a p<0.001 cutoff), and the light-yellow background indicates a modest significant overlap (p<0.05), while white background indicates not-significant (N.S.) overlap (p>0.05).

To investigate the roles of tomato SDG33 and SDG34 in regulating N responses, we compared transcriptomes of CRISPR mutants *sdg33* or *sdg34* (Bvindi et al., 2022) and wild type plants (WT) grown under N-supplied (+N) or N-starved (-N) conditions (**Figure 1A**). In detail, *sdg33* and *sdg34* mutants, as well as WT, were grown hydroponically in N-rich media for two weeks, followed by N starvation for 48 hours, and then transferred to media supplemented with N (+N) or control media without N (-N). After five-hour of N treatments, shoot and root tissues were harvested separately for RNA-Seq to determine the genome-wide regulatory effects of SDG33 and SDG34 in N responses. RNA-Seq reads were mapped to the tomato genome using BBmap (Bushnell et al., 2017) and then counted using FeatureCount (Liao et al., 2014). Differentially expressed genes (DEGs) were determined using DESeq2 with the design (~Genotype+Treatment+Genotype : Treatment).

We first identified DEGs that are affected by genotype (WT vs *sdg33* and WT vs *sdg34*) regardless of N conditions (statistical cut-offs: |log2(WT/mutant) | > 2; FDR < 5%). Overall, mutations in *sdg33* or *sdg34* lead to altered expression of hundreds of genes in the shoots and in the roots. In the shoots, 653 and 634 up-regulated genes, and 951 and 683 downregulated genes were identified in *sdg33* and *sdg34* separately (**Figure 1B**; **Supplemental Tables S1 & S2**). In the roots, 416 and 350 up-regulated genes, and 167 and 120 down-regulated genes were identified in *sdg33* and *sdg34* separately (**Figure 1C**; **Supplemental Table S3 & S4**). Since SDG33 and SDG34 proteins share 71% similarity in amino acid sequence, we investigated whether SDG33 and SDG34 have similar or diverged regulatory roles by comparing their downstream regulated genes. We observed highly significant overlaps between the DEGs regulated by SDG33 and SDG34, while they each regulate a smaller unique set of genes (number of overlapped and unique DEGs, and p-values for the overlaps were shown as Venn diagrams in **Figures 1B, C**). In the shoots, 479 up-regulated genes shared between *sdg33* and *sdg34* are enriched with GO terms “response to auxin” and “oxidoreductase activity”, indicating that both SDG33 and SDG34 repress genes involved in auxin response and oxidoreductase activity (**Supplemental Table S5**; **Figure 1B**). Interestingly, the 174 genes that are significantly repressed by SDG33 only also have the same significantly enriched GO terms “response to auxin” and “oxidoreductase activity” (**Supplemental Table S6**; **Figure 1B**). By contrast, the 155 genes that are uniquely repressed by SDG34 have different GO terms (**Figure 1B**). This suggests that the biological processes regulated by SDG33 are comparable to the “core” function shared between SDG33 and SDG34. This is also supported by the down-regulated genes identified in the shoots: both the 558 shared down-regulated DEGs and the 393 DEGs uniquely down-regulated in the *sdg33* mutant are significantly enriched with GO terms related to transcriptional regulation activity (**Supplemental Table S7**; **Figure 1B**). In the roots, 260 genes were commonly up-regulated in both *sdg33* and *sdg34* (**Figure 1C**), with significantly enriched GO terms “response to auxin” and “oxidation-reduction process” (**Supplemental Table S8**; **Figure 1C**), similar to those identified in the shoots (**Figure 1B**). Therefore, SDG33 and SDG34 repress genes involved in auxin response and oxidation-reduction process in both shoots and roots. In either shoots or roots, the up-regulated genes identified in one mutant do not overlap with the down-regulated genes in the other mutant, and *vice versa*.

Next, we asked if the regulatory targets of histone methyltransferases are conserved between species. Indeed, SDG33 and SDG34 activate genes involved in transcriptional regulation and primary metabolism in the shoots (**Figure 1B**), similar to their Arabidopsis ortholog SDG8 (Li et al., 2015). To further study this, we compared genes regulated by SDG33 and SDG34 in tomato with genes regulated by their Arabidopsis ortholog SDG8 (Li et al., 2015) through homology mapping (BLASTP with E-value < 1e-6). Interestingly, the down-regulated genes in *sdg33*, but not those in *sdg34*, have a significant overlap with down-regulated genes in the *sdg8* mutant in Arabidopsis (**Figure 1D**; significant overlap with p<0.001 as determined by Genesect in the VirtualPlant platform (Katari et al., 2010)). By contrast, the up-regulated genes in *sdg34*, compared to those in *sdg33*, have a more significant overlap with up-regulated genes in the *sdg8* mutant in Arabidopsis (**Figure 1D**). Therefore, our results suggested that SDG33 shares the activated targets with its Arabidopsis homolog SDG8, while SDG34 shares the repressed targets with Arabidopsis SDG8.

Overall, our results support that the tomato SDG33 and SDG34 share the majority of their downstream regulated genes, in addition to a subset of distinct target genes they each regulate. In the shoots, SDG33 and SDG34 activate genes involved in primary metabolism and transcriptional regulation. In the shoots as well as in the roots, SDG33 and SDG34 repress the expression of genes involved in auxin response and oxidation-reduction process. In addition to the shared “core” function, we observed a possible functional divergence between SDG33 and SDG34: SDG33 seems to preserve more of the core function shared between SDG33 and SDG34, while SDG34 regulates additional processes; in addition, through cross-species comparison of the regulatomes (the collection of genes transcriptionally regulated by SDG33/34), SDG33 shares the positively regulated targets with the Arabidopsis homolog SDG8, while SDG34 shares more of the negatively regulated targets.

### SDG33 and SDG34 mediate N-responsive gene regulatory networks in roots

We next analysed our RNA-Seq datasets to determine whether SDG33 and SDG34 mediate N-responsive gene regulations. To do this, we identified DEGs whose transcriptional regulation by N is dependent on SDG33 or SDG34, using DESeq2 with the model ~Genotype+Treatment+Genotype : Treatment and focused on genes affected by the interaction term Genotype : Treatment (*i.e. sdg33* x N or *sdg34* x N), in shoots and roots separately.

**Figure 2:**
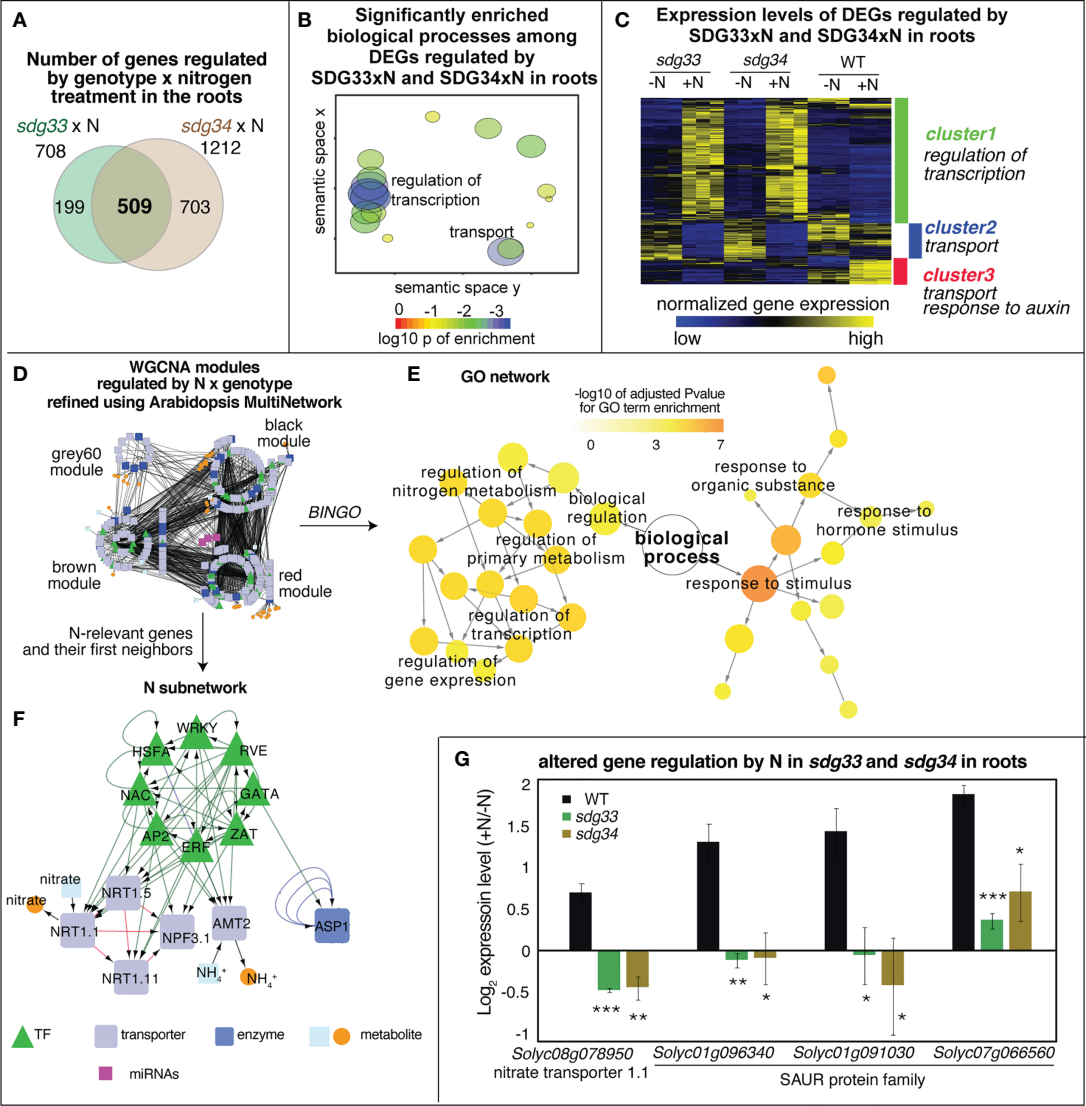
Differentially expressed genes (DEGs) regulated by the interaction of genotype and nitrogen (N) in roots. **(A)** Root transcriptomes were analyzed to identify DEGs that are regulated by the interaction of genotype and nitrogen, *i.e.*, DEGs whose nitrogen regulation is dependent on SDG33 and SDG34. 708 DEGs were identified for *sdg33* and 1212 DEGs were identified for *sdg34*, out of which 509 genes are shared between two mutants. **(B)** Significant GO terms such as “regulation of transcription” and “transport” were identified among these shared 509 DEGs, as shown in the semantic space of GO terms generated by ReviGO. Each circle in the sematic space represents a significant GO term, and the size and color of the circle represent the level of significance of the enrichment. **(C)** The expression patterns of these 509 shared DEGs are shown as a heatmap, which is clustered into three major clusters. **(D–F)** Network analyses of the shared DEGs have identified a network **(D)** enriched with GO terms such as “regulation of transcription”, “regulation of nitrogen metabolism”, and “response to stimulus” as determined by BINGO **(E)**. **(F)** An N-relevant subnetwork was extracted from the main network **(D)**, which highlights the known N transport and assimilation genes, and their interacting genes based on the network inference. In this N subnetwork, *NRT1.1* is the most regulated gene. **(G)** The expression regulation pattern of *NRT1.1*, measured as the log2 fold change of expression levels between +N and -N conditions, is shown together with the regulation patterns of three *SAUR* genes. P-values were determined by comparing WT and *sdg33* or *sdg34* using student t-tests: ***p<0.001; **p<0.01; *p<0.05.

In the roots, 708 DEGs were regulated by the interaction between the *sdg33* mutation and N treatments, and 1,212 DEGs were regulated by the interaction between the *sdg34* mutant and N treatments (**Figure 2A**). These two gene groups share 509 common genes (**Figure 2A**), which are significantly enriched with GO terms “transcriptional regulation” and “transmembrane transport” (**Figure 2B**, **Supplemental Table S10**), and form three major clusters (**Figure 2C**, **Supplemental Table S11**): (*i*) Cluster 1 comprises 343 genes that are induced by N treatment in the mutants but not in the WT (**Figure 2C**), enriched with GO term “regulation of transcription DNA template” (**Supplemental Table S12**); (*ii*) Cluster 2 comprises 100 genes whose expression is down-regulated in response to N in the mutants but to a less degree in the WT (**Figure 2C**), and is enriched with biological functions “transport” (**Supplemental Table S13**); and (*iii*) Cluster 3 contains 66 genes that are induced by N in the WT but are less responsive or even repressed in the mutants (**Figure 2C**). The cluster 3 genes are significantly enriched with biological processes “transport” and “response to auxin” (**Supplemental Table S14**). Given that SDG33 and SDG34 are required for depositing permissive marks H3K36me3 and H3K4me3 (Bvindi et al., 2022), it is likely that cluster 2 and 3 are enriched with the direct targets of SDG33/34.

To identify the most important misregulated genes, we investigated the regulatory hierarchy among the interaction DEGs by constructing gene regulatory networks. To do this, we first identified 89 co-expression gene network modules from our root RNA-Seq data using WGCNA (Langfelder and Horvath, 2008). We detected four modules, red, black, brown, and grey60, that are each significantly enriched with interaction DEGs. In total, 304 genes among these four modules belonged to the 509 interaction DEGs (**Supplemental Table S11**). We further inferred and refined the interactions among these 304 genes using known gene-to-gene interactions (Katari et al., 2010), which resulted in a network with the following characteristics (**Figure 2D**): (*i*) genes in this network are regulated by *sdg33*xN and *sdg34*xN; (*ii*) the network consists of four modules (grey60, black, brown, and red), and genes within each module shared co-expression relationships; (*iii*) for any gene pair connected by an edge, their Arabidopsis homologs have known gene-to-gene interactions, including protein-protein interactions (*e.g.* from BIND database (Alfarano et al., 2005)), or transcriptional regulation (*e.g.* AGRIS (Davuluri et al., 2003) or DAP-Seq (Bartlett et al., 2017)), based on the gene-to-gene interaction database Multinetwork hosted in the VirtualPlant platform (Katari et al., 2010). This network is enriched with genes involved in biological processes “response to stimulus” and “regulation of nitrogen metabolic process” as determined by BINGO analysis (Maere et al., 2005) (**Figure 2E**), suggesting that SDG33 and SDG34 control the N-responsive expression of a network of interacting genes involved in signaling and primary metabolism.

To focus on N metabolic genes, a subnetwork was extracted from the original network (**Figure 2D**) by filtering for known transporters and enzymes of N metabolism and their interacting partners (first neighbors) as previously performed (Para et al., 2014) (**Figure 2F**). It is worth noting that this N subnetwork inherited the gene-to-gene interaction relationships from the original network (**Figure 2D**) including the above-mentioned co-expression relationships and curated gene-to-gene interactions of Arabidopsis homologs. This N-subnetwork consists of 14 genes involved in the sensing, uptake, and assimilation of N, including many transporters: three nitrate transporters, *NRT1.1*, *NRT1.5*, and *NRT1.11*, one ammonium transporter (*AMT2*), and one nitrate transporter family protein (*NPF3.1*) (**Figure 2F**). The most regulated gene in this subnetwork encodes nitrate transporter *NRT1.1* (*Solyc08g078950.3*), a homolog to Arabidopsis *AtNRT1.1* (*AT1G12110*). In our study, the N-responsive regulation of *NRT1.1* is dependent on SDG33/34. *NRT1.1* is induced by a supply of N in the WT, but not in *sdg33* or *sdg34* mutants (**Figure 2G**). The Arabidopsis homolog AtNRT1.1 has dual transporter activity for auxin and nitrate: in the absence of nitrate, NRT1.1 transport auxin shootward; in the presence of nitrate, NRT1.1 transports nitrate, which results in an altered auxin pattern that stimulates the growth of lateral roots (Krouk et al., 2010; Sun and Zheng, 2015). Therefore, the misregulation of *NRT1.1* in the mutants indicates that N uptake and auxin distribution patterns in the roots are possibly altered when histone methyltransferase SDG33/34 is mutated. In agreement with this, the GO term “response to auxin” is significantly enriched among the cluster 3 genes that are N responsive in WT but misregulated in the mutants (**Figure 2C**). This cluster includes three SAUR-like auxin-responsive genes (Ren and Gray, 2015) (*Solyc01g096340*, *Solyc01g091030*, and *Solyc07g066560*) that are responsive to N in the WT, but are unchanged, repressed, or induced to a lesser degree in the *sdg33* or *sdg34* mutant (**Figure 2G**). The SAUR gene family has been well characterized for their roles in mediating auxin-triggered cell elongation through modulating the acidity of cell walls (Stortenbeker and Bemer, 2019). Overall, our root transcriptome analysis indicates a widespread misregulation of genes in response to N in the *sdg33* and *sdg34* mutants compared to WT. Moreover, the misregulation of *NRT1.1* and *SAURs* collectively linked SDG33 and SDG34 with auxin signaling and lateral root growths during N response.

### Mutations in *sdg33* or *sdg34* alter N-responsive root development

**Figure 3:**
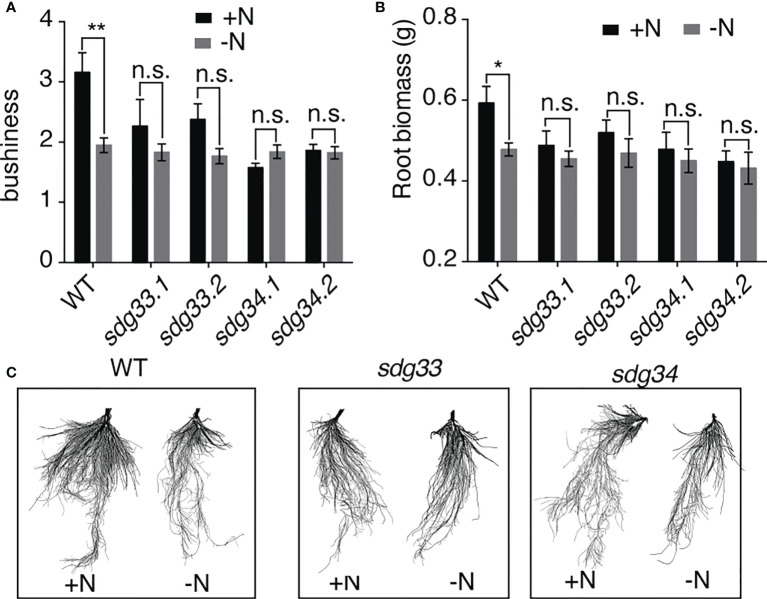
Root response to nitrogen requires SDG33 and SDG34. **(A, B)** In response to a supply of nitrogen, the bushiness that reflects the expansion of lateral roots **(A)** and root biomass **(B)** of WT plants are induced, but these responses are abolished in the *sdg33* and *sdg34* mutants. **(C)** Representative photos of the roots of WT, *sdg33*, and *sdg34* are shown. P-values were determined by comparing the root measurements between WT and *sdg33* or *sdg34* using student t-tests: **p<0.01; *p<0.05.

Informed by the root transcriptomic analysis, we tested the hypothesis that SDG33 and SDG34 regulate lateral root growth in response to N supply. To do this, *sdg33* and *sdg34* mutants, including two independent lines for each mutant, as well as WT plants were treated with N conditions (-N and +N) as previously performed for the transcriptomic analysis. The root architecture was compared using GiARoots (Galkovskyi et al., 2012) after seven days of N treatments, since physiological changes take longer time to observe than gene expression level changes which occur in hours. Interestingly, the lateral growth of the root system (measured as bushiness *i.e.* the ratio of the maximum number of roots divided by the minimum number of roots along the vertical axis of roots) was increased significantly by the N supply in the WT, but not in the *sdg33* or *sdg34* mutants (**Figure 3A**), which is also shown by root images (**Figure 3C**). This result was further supported by measuring root biomass (**Figure 3B**), which showed a significant root growth induced by a N supply in WT but not in mutants. Overall, our results suggest that N stimulates root growth in the WT but not in the mutants where the histone methyltransferases SDG33 or SDG34 are mutated. This possibly involves misregulation of auxin signaling pathways in the *sdg33* and *sdg34* mutants, as SDG33/34 regulates *NRT1.1* and multiple *SAUR* genes (**Figure 2**), while auxin-independent mechanisms are also possible.

### SDG33 and SDG34 affect the expression of photosynthetic genes in response to N changes in shoots

**Figure 4:**
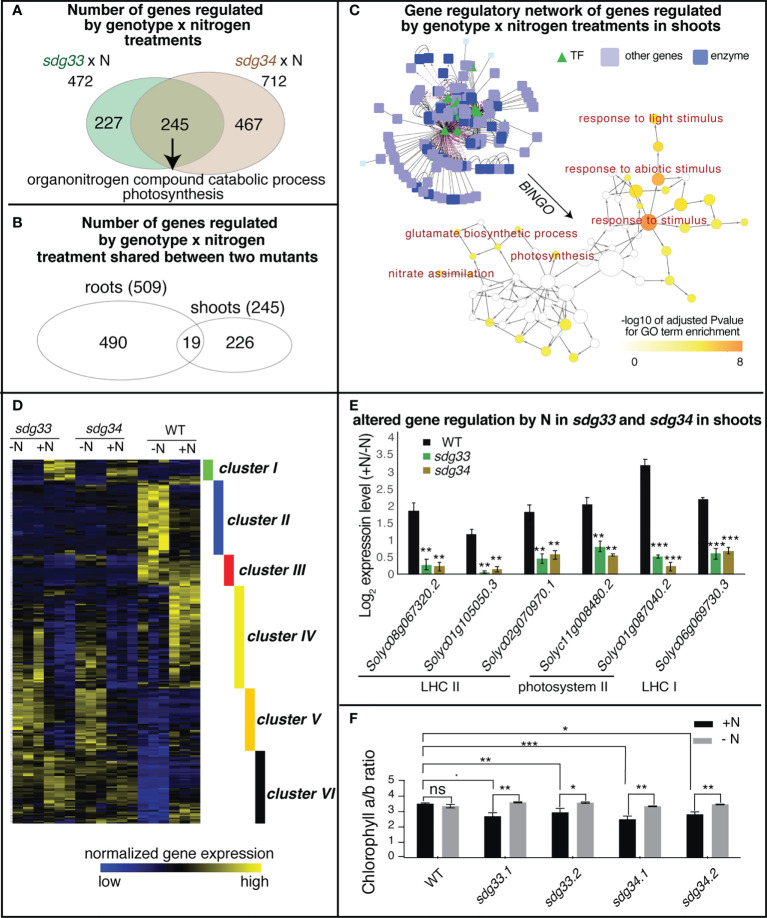
Differentially expressed genes (DEGs) regulated by the interaction of genotype and nitrogen in shoots. **(A)** Shoot transcriptomes were analyzed to identify DEGs that are regulated by the interaction of genotype and nitrogen, *i.e.* DEGs whose nitrogen regulation is dependent on SDG33 and SDG34. 472 DEGs were identified for *sdg33* and 712 DEGs were identified for *sdg34*, out of which 245 genes are shared between two mutants. **(B)** These shared 245 DEGs identified in the shoots have a modest overlap with that identified in the roots, suggesting that the SDG33/34 dependent regulation of genes by nitrogen is highly organ-specific. **(C)** Network analyses of the shared DEGs have identified a network enriched with GO terms such as “response to stimulus” and “photosynthesis” as determined by BINGO. **(D)** The expression patterns of these shared 245 DEGs identified in the shoots are shown as a heatmap and are clustered into six groups. **(E)** Six genes involved in photosystems are misregulated in the mutants compared to WT. The log 2 fold change of gene expression levels between +N and -N conditions were plotted and p-value was determined by student’s t-test of between mutants and WT: ****p<0.0001; ***p<0.001; **p<0.01; *p<0.05.; and ˙: p<0.1. **(F)** chlorophyll a to b ratio is altered in the *sdg33* and *sdg34* mutants compared to WT when nitrogen is provided. P-values determined by student’s t-tests: ***p<0.001; **p<0.01; *p<0.05; and ˙p<0.1.

In the shoots, 472 and 712 genes were regulated by the interaction between SDG33 or SDG34 and N treatments (*sdg33* x N or *sdg34* x N), separately. Of these, 245 genes were commonly shared between two mutants, enriched with GO terms “organonitrogen compound catabolic process” and “photosynthesis” (**Figure 4A**; **Supplemental Table S15**). There is only modest overlap between the interaction DEGs identified in shoots and those identified in roots, indicating that the SDG33/SDG34 mediated N-regulation is largely dependent on organ context (**Figure 4B**).

From the 245 shared interaction DEGs in the shoots, a gene regulatory network was constructed using the known gene-to-gene interactions of their Arabidopsis homologs, including protein-protein interactions (*e.g.* based on BIND database (Alfarano et al., 2005)) and transcriptional regulation (*e.g.* AGRIS (Davuluri et al., 2003) or DAP-Seq (Bartlett et al., 2017)), from the Multinetwork database in the VirtualPlant platform (Katari et al., 2010). Collectively, this gene network is enriched with biological processes such as response to stimulus especially response to light stimulus, and primary metabolic pathways including both nitrate and amino acid metabolism and photosynthesis (**Figure 4C**). To understand how these genes are regulated by SDG33/34 and N, the interaction DEGs were further classified into six clusters based on their gene expression patterns (**Figure 4D**; **Supplemental Table S16**). Among these, clusters II, IV, V, and VI are the bigger clusters comprising 86% of the DEGs. Cluster II genes, enriched with the GO term “response to abiotic stimulus” and “cellular amino acid catabolic process” (**Supplemental Table S17**), are induced by N starvation in the WT but not in either of the mutants, indicating that nutrient stress responses are misregulated in the mutants. Clusters IV and V genes, enriched with GO terms related to lipid metabolism and cell wall metabolism, respectively, display expression patterns that are induced by N in the WT but repressed by N in the mutants (**Supplemental Table S17**), possibly indicating that N-triggered cell growth is misregulated in the mutants. Cluster VI, which contains genes that are up-regulated by N supply in the WT but not in the mutants, has the highest significantly enriched GO term: “photosynthesis” (FDR<4E-6) (**Supplemental Table S17**). This result may suggest that while the WT plants show lower level of transcription of photosynthesis genes under -N condition and tune them up when N is resupplied, the *sdg33* or *sdg34* mutants lose the ability to respond.

Overall, the GO term enrichment analyses, no matter from the whole set of DEGs (**Figure 4A**), from the network analysis (**Figure 4C**), or through clustering (**Figure 4D**), have all highlighted the relevance of photosynthesis to N responses and SDG33/34 regulation. Indeed, Cluster VI includes genes encoding different photosynthetic components: three genes encoding subunits of Light Harvesting Complex II (LHC II: *Solyc08g067320*, *Solyc01g105050*, *Solyc02g070970*), two genes encoding subunits of photosystem II (PSII: *Solyc11g008480* and *Solyc01g087040*), and one gene related to LHC I (*Solyc06g069730*) (**Supplemental Table S16**; **Figure 4E**). Since different photosynthetic components have different chlorophyll a/b ratios, and N status was reported to change the relative amount or the organization of photosynthetic components to achieve carbon-nitrogen balance (Hikosaka and Terashima, 1995; Kitajima and Hogan, 2003), we tested if chlorophyll a/b is altered in the *sdg33* and *sdg4* mutants compared to WT in response to N changes (Khalyfa et al., 2002). Interestingly, in the *sdg33* and *sdg4* mutants, the chlorophyll a/b ratio is reduced compared to WT after a supply of N (**Figure 4F**). While WT plants showed stable chlorophyll a/b ratio when N level changes, the mutants displayed reduced chlorophyll a/b ratio after N supply (**Figure 4F**). Moreover, the mutants showed more fluctuation in non-regulated energy dissipation compared to WT in response to N level changes (**Figure S2A**). By contrast, the fresh weight of the aerial parts and the total chlorophyll content show no significant difference between WT and mutants (**Figure S2B, C**). Overall, our data suggest that SDG33 and SDG34 mediate genome-wide transcriptomic changes including photosynthetic genes in response to N in shoots. Specifically, SDG33 and SDG34 probably fine-tune photosynthetic apparatus and modulate the ratio of different light harvesting components in response to changing N environments.

## Discussion

Histone methylation has been shown to mediate plant response to environmental stress through modulating transcriptional regulation of functionally relevant genes (Li et al., 2015; Lee et al., 2016; Pandey et al., 2016; Li et al., 2019). While a handful of histone methyltransferases have been characterized in the model plant Arabidopsis (Ebbs et al., 2005; Ding et al., 2007; Tamada et al., 2009; Berr et al., 2010; Ding et al., 2011; Pan, 2013; Li et al., 2015; Liu et al., 2016), the function of histone methyltransferases in horticultural crops such as tomato has rarely been determined. In this study, we show that two tomato paralogous genes *SDG33* and *SDG34* encode histone methyltransferases with overlapping yet distinct functions and that mutations in *sdg33* and *sdg34* result in misregulation of N-responsive gene networks, which in turn results in altered physiological N response in tomato roots and shoots.

In Arabidopsis, histone methyltransferases were reported to modulate gene expression levels genome-wide (Makarevich et al., 2006; Kim et al., 2008; Tamada et al., 2009; Ding et al., 2012; Pan, 2013; Zhao et al., 2015; Li et al., 2015; Lee et al., 2016). Consistent with this, our transcriptome analysis revealed that tomato SDG33 and SDG34 are required for the proper expression of hundreds of genes in the shoots and roots (**Figure 1**), likely through modulating and maintaining the permissive chromatin status of their directly target genes, which in turn affects the expression levels of other downstream genes in the regulatory network. It is interesting to note that although they are implicated in depositing permissive histone marks H3K4me3 and H3K36me3 (Bvindi et al., 2022), SDG33 and SDG34 seem to also repress the expression of a group of genes while activating the expression of another group of genes in our transcriptomic analysis. It is possible that the down-regulated genes in the *sdg33* or *sdg34* mutants (thus gene targets activated by SDG33 or SDG34) are enriched with the direct targets of SDG33 and SDG34, as previously described for their Arabidopsis homolog SDG8 (Li et al., 2015), while the up-regulated genes in the mutants (*i.e.* downstream genes repressed by SDG33 or SDG34) are normally inhibited by intermediate repressors that are activated by SDG33/SDG34. It is also noteworthy that while H3K36me3 and the corresponding histone methyltransferases are well known to be associated with actively transcribed genes, the real image could be more complicated. For example, H3K36me3 was proposed to contribute to the transcriptional silencing of heterochromatin regions in a study (Chantalat et al., 2011). In addition, the mammalian homology of AtSDG8, SETD2, has a role in maintaining repressed chromatin in transcribed regions by recruiting a series of repressive epigenetic machinery (Schuhmacher et al., 2020). Therefore, we do not exclude the possibility that SDG33/34 may function to directly repress some of their gene targets. Alternatively, it is also plausible that loss of SDG33 or SDG34 leads to an increase of other histone modifications at certain gene loci that contribute toward the induction of these genes. For instance, in Arabidopsis, reduction of H3K36me3 in the *sdg8* mutant is linked to an increased level of H3K36ac, which is associated with gene activation (Mahrez et al., 2016). ChIP-sequencing assays with transgenic lines carrying epitope-tagged SDG33 and SDG34 would help to elucidate whether SDG33 or SDG34 directly or indirectly regulate the downstream genes whose expression is altered in the *sdg33* or *sdg34* mutants.

SDG33 and SDG34 share 71% similarity in protein sequence and 100% similarity in protein architecture (Aiese Cigliano et al., 2013). In agreement with this, we observed in our study that SDG33 and SDG34 share a significant number of overlapping target genes (**Figure 1**). However, there remains a portion of genes that are uniquely regulated by SDG33 or SDG34 showing that these two paralogs also perform different functions in addition to their shared regulatory roles. This is further supported by the different GO terms enriched among the genes uniquely regulated by SDG33 or SDG34. Interestingly, the GO terms enriched among the genes uniquely regulated by SDG33, but not those by SDG34, are similar to the GO terms enriched among the target genes shared by both. This result likely suggests that while SDG33 keeps the ancestral function, SDG34 has evolved to regulate new biological processes. This is also supported by the cross-species comparison of SDG33/34 regulatome in tomato with that in Arabidopsis. The Arabidopsis SDG8 was shown to mostly activate its direct targets (Li et al., 2015). In tomato, we found that SDG33, but not SDG34, shares a significant portion of the activated gene targets with SDG8 (**Figure 1D**).

It remains an intriguing question how SDG33 and SDG34 recognize their gene targets. It has been proposed that the target specificity of the Arabidopsis SDG8 was possibly determined by their CW domain which is able to bind methylated H3K4 (Liu and Huang, 2018). The CW domain is conserved in tomato SDG33 and SDG34 and could contribute toward the target specificity. It was also proposed that SDG8 identify targets by interacting with transcription factors (Li et al., 2015). How do SDG33 and SDG34 recognize different targets? One possibility is that the spatiotemporal expression of SDG33 and SDG34 might be different leading to different interactions with their downstream targets. We found this less supported because the expression patterns of SDG33 and SDG34 across different tissue types and treatment conditions are largely comparable (Bvindi et al., 2022). Another probable scenario is that SDG33 and SDG34 interact with different protein partners which leads to distinct target specificity, which could be tested using immunoprecipitation-Mass Spectrometry (IP-MS) with transgenic lines expressing epitope-tagged SDG33 or SDG34 proteins.

Histone methylations are known to mediate various environmental responses (Kim et al., 2008; Chinnusamy and Zhu, 2009; van Dijk et al., 2010; Jaskiewicz et al., 2011; Pan, 2013). Similar to our previous study in Arabidopsis (Li et al., 2019), mutations in *sdg33* and *sdg34* result in misregulation of N-responsive gene networks, which in turn results in altered physiological N response in tomato (**Figures 3**, **4**). Many genes seem to be dependent on SDG33 or SDG34 to remain transcriptionally responsive to N level changes (**Figures 3**, **4**), therefore, it is likely that SDG33 and SDG34 function to set a permissive chromatin context to allow transcriptional induction in response to environmental cues. While the previous Arabidopsis study only examined the aerial tissues, our current study in tomato provided a different perspective to examine the epigenetic regulation of N responses in roots, the major organ where N is sensed and taken up. Indeed, genes whose N-responsive expression is dependent on SDG33 and SDG34 are vastly different between shoots and roots, indicating a clear influence of organ context on chromatin regulation (**Figure 4B**). In the shoots, the altered N responses in *sdg33* and *sdg34* mutants highlighted genes related to primary metabolism, especially photosynthesis, similar to its Arabidopsis homolog SDG8 (Li et al., 2015; Li et al., 2020). At the physiological level, it is reflected by an altered chlorophyll a/b ratio in mutants compared to WT in an N-dependent manner (**Figure 4**), possibly suggesting that SDG33 and SDG34 are responsible for controlling the relative amount of different light harvesting components, *e.g.* stoichiometry of LHCs to reactions centers, in response to N supply. In line with this, we observed an increased NPQ-independent non-regulated energy loss in the mutants compared to WT under N-supplied conditions, possibly caused by the imbalance in light harvesting components in the mutants (**Supplemental Figure S2**). In the roots, DEGs regulated by the interaction of genotype and N are enriched with GO terms “regulation of transcription”, “transport”, “and response to auxin” (**Figure 2B, C**). Network analysis of these root genes led to the identification of a small subnetwork of genes (**Figure 2F**) that are: *i*) known N transporters and assimilation enzymes or their predicted regulators, and *ii*) regulated by N in an SDG33/34 dependent manner. The regulated hub of this network encodes the tomato homolog of the well-studied Arabidopsis nitrate transporter *NRT1.1*. In addition to functioning as a major nitrate transporter, Arabidopsis *NRT1.1* is essential for plants to sense the availability of nitrate and to stimulate lateral root growth to colonize N-rich soil patches (Remans et al., 2006). Our data shows that in the WT tomato, *NRT1.1* is induced by a supply of N (**Figure 2G**), which is in agreement with increased root growth in N-supplied conditions compared to N-deprived condition observed in WT (**Figure 3**). This N-mediated regulation of *NRT1.1* is abrogated in the *sdg33* or *sdg34* mutants (**Figure 2G**), which is associated with a loss of the root response to the N treatments (**Figure 3**). This suggests that the tomato homolog of *NRT1.1* is likely also involved in sensing nitrate and stimulating root growth; furthermore, our study in tomato now uncovered that the N-responsive regulation of *NRT1.1* and root growth requires functional SDG33 and SDG34.

In Arabidopsis, *NRT1.1*-mediated lateral root growth has been connected to auxin transport and signaling (Krouk et al., 2010; Bouguyon et al., 2015): in the absence of nitrate, NRT1.1 facilitates basipetal transport of auxin, thus lowering auxin accumulation in the lateral root tips, as a result, lateral root growth and elongation are limited; By contrast, under high $NO_{3}^{-}$ condition, the auxin transport by NRT1.1 is inhibited, allowing auxin to accumulate and stimulate lateral root growth. In agreement with this, our transcriptomic analysis in the roots identified “response to auxin” as a significant GO term enriched among the DEGs regulated by N x SDG33 or N x SDG34. This includes a group of *SMALL AUXIN-UPREGULATED RNA (SAUR)* genes (**Figures 2C, G**), which has been previously reported to be induced by auxin and in turn promote cell expansion and growth through protein phosphorylation and apoplastic acidification (Spartz et al., 2014). In our study, the *SAUR* genes are induced by N in the WT but not in the mutants, suggesting that the N-induction of these *SAUR* gene family require SDG33 and SDG34. Altogether, we speculate the following plausible mechanism for chromatin level regulation of N-induced lateral root growth (**Supplemental Figure S3**): in the WT, when $NO_{3}^{-}$ is sufficient, the expression of nitrate transporter *NRT1.1* is induced, through SDG33 and SDG34 directly or indirectly, and the auxin transport function of NRT1.1 protein is inhibited, resulting in auxin accumulation at the lateral root tips. This in turn induces the expression of *SAUR* genes, either directly or indirectly through SDG33/34, to promote cell expansion, which leads to lateral root growth. In the *sdg33* and *sdg34* mutants, the genes *NRT1.1* and *SAURs* are misregulated, therefore leading to the loss of root plasticity responsive to N changes (**Figures 3**, **S3**). It is worth noting that it is yet to be proved that the tomato homolog of *NRT1.1* function in a similar manner to its Arabidopsis homolog as a transporter for both nitrate and auxin, and that future study is needed to determine whether *NRT1.1* and the *SAURs* are direct or indirect targets of SDG33 and SDG34.

Moreover, the Arabidopsis ortholog SDG8 is known to mediate plant defense (Berr et al., 2010; Lee et al., 2016) as well as plant response to N supply (Li et al., 2019). As previously mentioned, plant N status was shown to affect its defense responses (Verly et al., 2020; Soulie et al., 2020). Therefore, it is possible the SDG8 functions as the regulatory hub of the crosstalk of N response and pathogen response. In support of this, SDG8 was shown to directly regulate *NON-EXPRESSOR OF PATHOGENESIS-RELATED GENES 1* (Zhang et al., 2020), which mediates N-dependent defense response (Verly et al., 2020). The *sdg33/sdg34* tomato mutants showed altered pathogen response (Bvindi et al., 2022), and now this study suggested that SDG33 and SDG34 also mediate N responses in tomato. It will be of future interest to test if SDG33 and SDG34 (and Arabidopsis SDG8) function to integrate N environment and pathogen defense in plants.

In summary, our study demonstrated that the two paralogous histone methyltransferases SDG33 and SDG34 play overlapping yet distinct roles in regulating gene expression in tomato. SDG33 and SDG34 are both required for N responses in tomato, regulating organ-specific gene networks in the roots and shoots. We uncovered that in the roots SDG33 and SDG34 mediate N-regulation of *NRT1.1* and lateral root growth, possibly involving the auxin signaling pathway. We also uncovered the role of SDG33 and SDG34 in maintaining the chlorophyll a to b ratio in the leaves when the N level changes. Mechanistic insights into the determinants of the organ specificity and target specificity for histone methylation enzymes will be of general interest to further the understanding of the mode-of-action of chromatin regulation in mediating plant responses to the environment.

## Data availability statement

The original contributions presented in the study are publicly available. This data can be found here: NCBI GEO, GSE195546.

## Author contributions

YL and TM conceived the project; CB, SL, RMP and ZRY performed the experiments; YL, CB and LT analyzed the data; CB and YL wrote the article with contributions of all the authors; YL agrees to serve as the author responsible for contact and ensures communication. All authors contributed to the article and approved the submitted version.

## Funding

This work is supported by the Purdue Startup fund to YL, Fulbright fellowship to CB, Purdue Center for Plant Biology Graduate student fellowship to LT, National Science Foundation (IOS-1916893) to TM, and National Science Foundation (MCB-2123470) and USDA National Institute of Food and Agriculture Hatch fund (Accession number 1013620) to YL.

## Acknowledgments

We would like to thank Dr. Matthew Brooks (USDA ARS) for insightful discussion of the data.

## Conflict of interest

The authors declare that the research was conducted in the absence of any commercial or financial relationships that could be construed as a potential conflict of interest.

## Publisher’s note

All claims expressed in this article are solely those of the authors and do not necessarily represent those of their affiliated organizations, or those of the publisher, the editors and the reviewers. Any product that may be evaluated in this article, or claim that may be made by its manufacturer, is not guaranteed or endorsed by the publisher.
