# Supplementary material for: Histone methyltransferases SDG33 and SDG34 regulate organ-specific nitrogen responses in tomato
Source: Front Plant Sci. 2022 Oct 12;13:1005077. doi: 10.3389/fpls.2022.1005077 (PMC9606235; doi:10.3389/fpls.2022.1005077)
Supplement: Supplementary file 1 [file DataSheet_1.pdf]

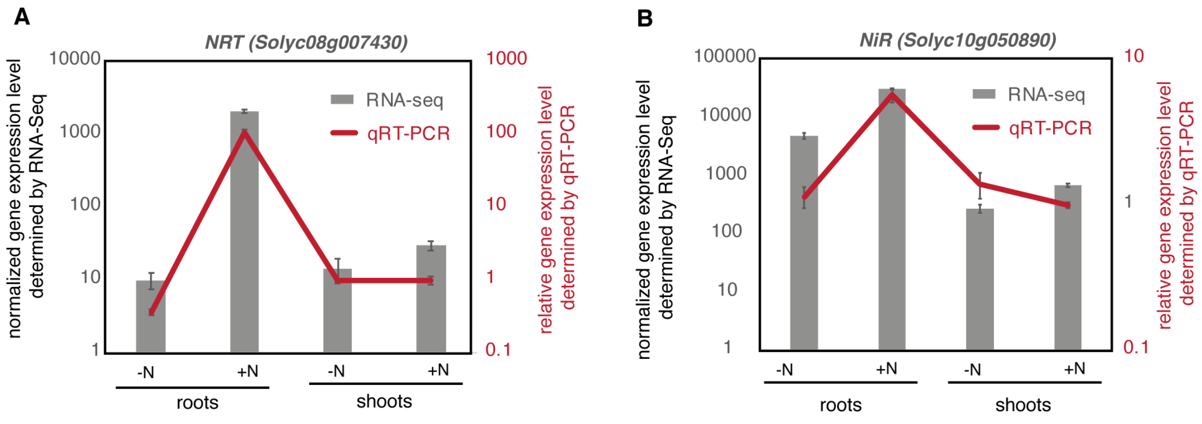

Supplemental Figure S1. Gene expression levels determined by RNA-seq (grey block, Primary Y axis) and qRT-PCR (red line, secondary Y axis) are comparable. The qRT-PCR was performed and analyzed as previously described by Bvindi et al., 2022, with the *SlActin* being used as an internal control. The error bars represent mean of standard errors.

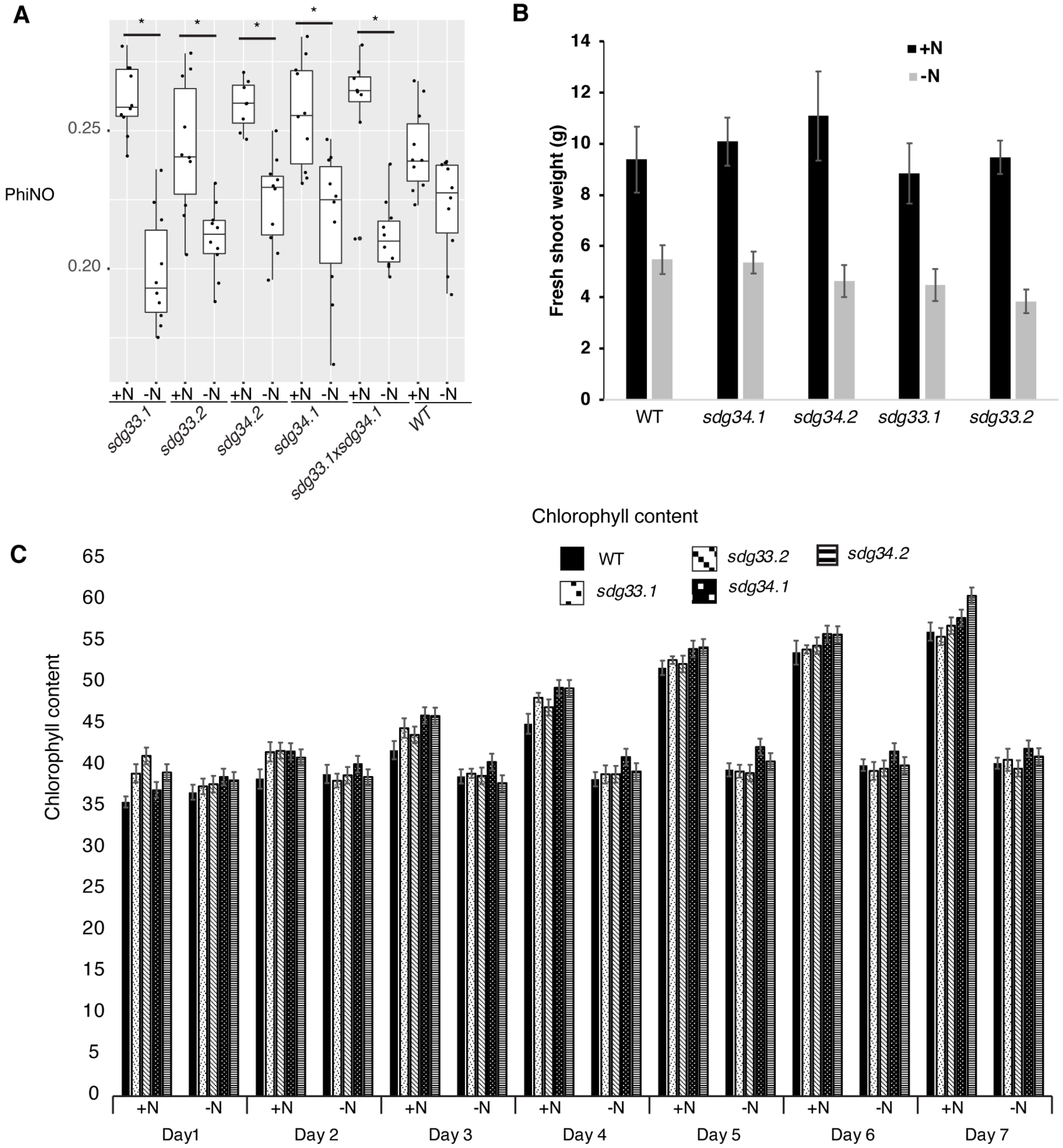

Supplemental Figure 2. PhiNO (A) shows bigger fluctuation in response to N in the mutants compared to WT. P-values are determined by two-way ANOVA (genotype:N interaction term): ****: $p<0.0001$; ***: $p<0.001$; **: $p<0.01$; *: $p<0.05$; and ˙: $p<0.1$. Fresh shoot weight (B) and total chlorophyll (C) are not significantly different between the *sdg33* and *sdg34* mutants compared to WT, while they both show great increase when nitrogen is provided.

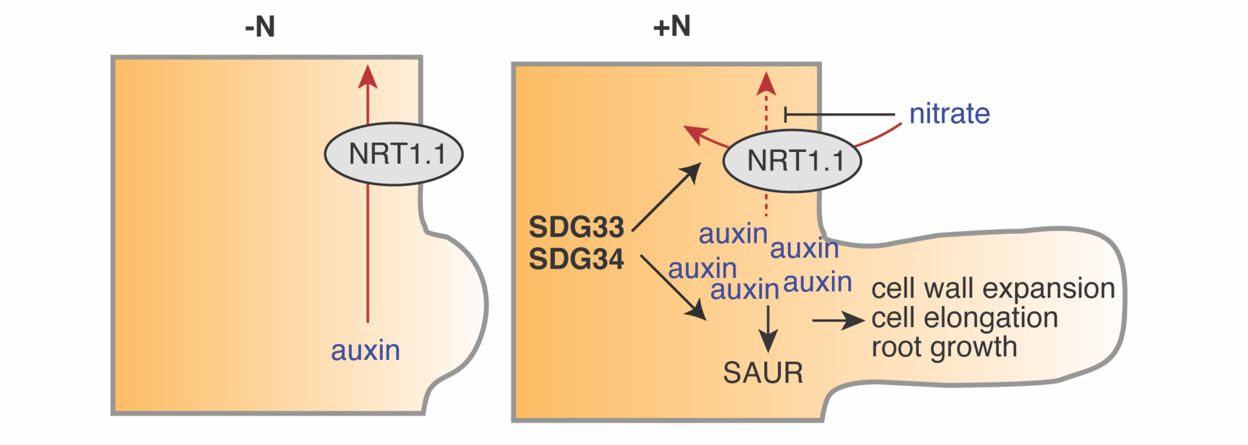

Supplemental Figure S3. The proposed model of the role of SDG33 and SDG34 in mediating nitrate induced root growth.
